# AI4People—An Ethical Framework for a Good AI Society: Opportunities, Risks, Principles, and Recommendations

**DOI:** 10.1007/s11023-018-9482-5

**Published:** 2018-11-26

**Authors:** Luciano Floridi, Josh Cowls, Monica Beltrametti, Raja Chatila, Patrice Chazerand, Virginia Dignum, Christoph Luetge, Robert Madelin, Ugo Pagallo, Francesca Rossi, Burkhard Schafer, Peggy Valcke, Effy Vayena

**Affiliations:** 1 Oxford Internet Institute, University of Oxford, Oxford, UK; 2 The Alan Turing Institute, London, UK; 3 Naver Corporation, Grenoble, France; 4 French National Center of Scientific Research, Paris, France; 5 Institute of Intelligent Systems and Robotics, Pierre and Marie Curie University, Paris, France; 6 Digital Europe, Brussels, Belgium; 7 University of Umeå, Umeå, Sweden; 8 Delft Design for Values Institute, Delft University of Technology, Delft, The Netherlands; 9 TUM School of Governance, Technical University of Munich, Munich, Germany; 10 Centre for Technology and Global Affairs, University of Oxford, Oxford, UK; 11 Department of Law, University of Turin, Turin, Italy; 12 IBM Research, New York, USA; 13 University of Padova, Padua, Italy; 14 University of Edinburgh Law School, Edinburgh, UK; 15 Centre for IT & IP Law, Catholic University of Leuven, Flanders, Belgium; 16 Bocconi University, Milan, Italy; 17 Bioethics, Health Ethics and Policy Lab, ETH Zurich, Zurich, Switzerland

**Keywords:** Artificial intelligence, AI4People, Data governance, Digital ethics, Governance, Ethics of AI

## Abstract

This article reports the findings of AI4People, an Atomium—EISMD initiative designed to lay the foundations for a “Good AI Society”. We introduce the core opportunities and risks of AI for society; present a synthesis of five ethical principles that should undergird its development and adoption; and offer 20 concrete recommendations—to assess, to develop, to incentivise, and to support good AI—which in some cases may be undertaken directly by national or supranational policy makers, while in others may be led by other stakeholders. If adopted, these recommendations would serve as a firm foundation for the establishment of a Good AI Society.

## Introduction

AI is not another utility that needs to be regulated once it is mature. It is a powerful force, a new form of smart agency, which is already reshaping our lives, our interactions, and our environments. AI4People was set up to help steer this powerful force towards the good of society, everyone in it, and the environments we share. This article is the outcome of the collaborative effort by the AI4People Scientific Committee—comprising 12 experts and chaired by Luciano Floridi^1^—to propose a series of recommendations for the development of a Good AI Society.

The article synthesises three things: the *opportunities* and associated *risks* that AI technologies offer for fostering human dignity and promoting human flourishing; the *principles* that should undergird the adoption of AI; and 20 specific *recommendations* that, if adopted, will enable all stakeholders to seize the opportunities, to avoid or at least minimise and counterbalance the risks, to respect the principles, and hence to develop a Good AI Society.

The article is structured around four more sections after this introduction. Section 2 states the core opportunities for promoting human dignity and human flourishing offered by AI, together with their corresponding risks.^2^ Section 3 offers a brief, high-level view of the advantages for organisations of taking an ethical approach to the development and use of AI. Section 4 formulates 5 ethical principles for AI, building on existing analyses, which should undergird the ethical adoption of AI in society at large. Finally, Sect. 5 offers 20 recommendations for the purpose of developing a Good AI Society in Europe.

Since the launch of AI4People in February 2018, the Scientific Committee has acted collaboratively to develop the recommendations in the final section of this paper. Through this work, we hope to have contributed to the foundation of a Good AI Society we can all share.

## The Opportunities and Risks of AI for Society

That AI will have a major impact on society is no longer in question. Current debate turns instead on how far this impact will be positive or negative, for whom, in which ways, in which places, and on what timescale. Put another way, we can safely dispense with the question of *whether* AI will have an impact; the pertinent questions now are *by whom, how*, *where*, and *when* this positive or negative impact will be felt.

**Fig. 1:**
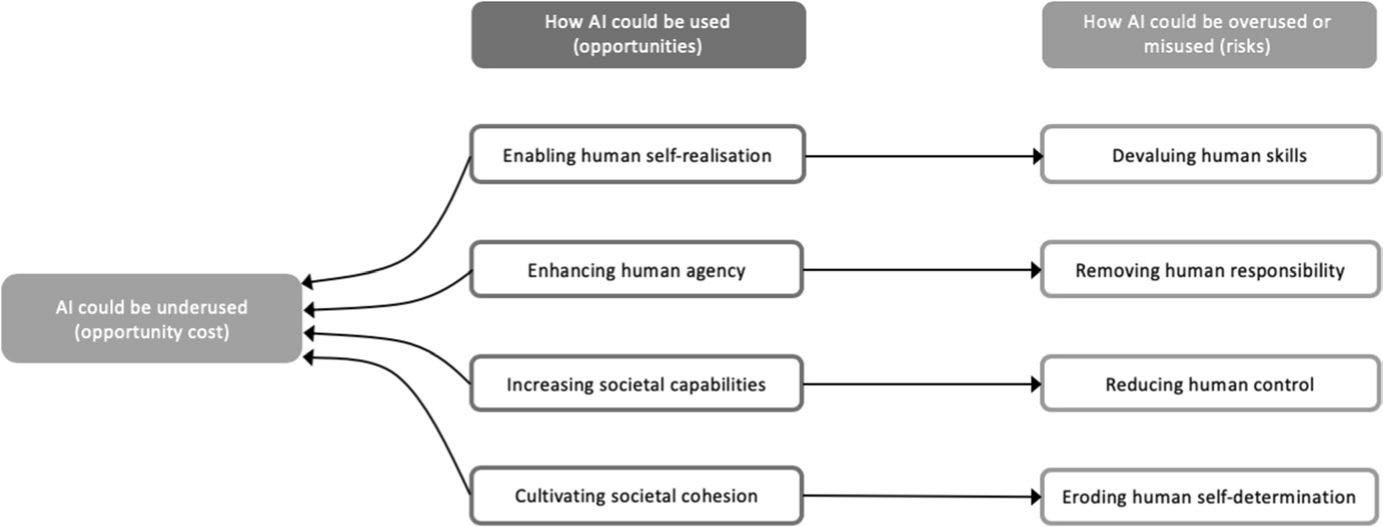
Overview of the four core opportunities offered by AI, four corresponding risks, and the opportunity cost of underusing AI

In order to frame these questions in a more substantive and practical way, we introduce here what we consider the four chief opportunities for society that AI offers. They are four because they address the four fundamental points in the understanding of human dignity and flourishing: *who we can become* (autonomous self-realisation); *what we can do* (human agency); *what we can achieve* (individual and societal capabilities); and *how we can interact with each other and the world* (societal cohesion). In each case, AI can be *used* to foster human nature and its potentialities, thus creating opportunities; *underused*, thus creating opportunity costs; or *overused* and *misused*, thus creating risks. As the terminology indicates, the assumption is that the *use* of AI is synonymous with good innovation and positive applications of this technology. However, fear, ignorance, misplaced concerns or excessive reaction may lead a society to *underuse* AI technologies below their full potential, for what might be broadly described as the wrong reasons. This may cause significant opportunity costs. It might include, for example, heavy-handed or misconceived regulation, under-investment, or a public backlash akin to that faced by genetically modified crops (Imperial College 2017). As a result, the benefits offered by AI technologies may not be fully realised by society. These dangers arise largely from unintended consequences and relate typically to good intentions gone awry. However, we must also consider the risks associated with inadvertent *overuse* or wilful *misuse* of AI technologies, grounded, for example, in misaligned incentives, greed, adversarial geopolitics, or malicious intent. Everything from email scams to full-scale cyber-warfare may be accelerated or intensified by the malicious use of AI technologies (Taddeo 2018). And new evils may be made possible (King et al. 2018). The possibility of social progress represented by the aforementioned opportunities above must be weighed against the risk that malicious manipulation will be enabled or enhanced by AI. Yet a broad risk is that AI may be underused out of fear of overuse or misuse. We summarise these risks in Fig. 1 below, and offer a more detailed explanation in the text that follows.

### Who We Can Become: Enabling Human Self-Realisation, Without Devaluing Human Abilities

AI may enable self-realisation, by which we mean the ability for people to flourish in terms of their own characteristics, interests, potential abilities or skills, aspirations, and life projects. Much as inventions, such as the washing machine, liberated people—particularly women—from the drudgery of domestic work, the “smart” automation of other mundane aspects of life may free up yet more time for cultural, intellectual and social pursuits, and more interesting and rewarding work. More AI may easily mean more human life spent more intelligently. The risk in this case is not the obsolescence of some old skills and the emergence of new ones per se, but the pace at which this is happening and the unequal distributions of the costs and benefits that result. A very fast devaluation of old skills and hence a quick disruption of the job market and the nature of employment can be seen at the level of both the individual and society. At the level of the individual, jobs are often intimately linked to personal identity, self-esteem, and social role or standing, all factors that may be adversely affected by redundancy, even putting to one side the potential for severe economic harm. Furthermore, at the level of society, the deskilling in sensitive, skill-intensive domains, such as health care diagnosis or aviation, may create dangerous vulnerabilities in the event of AI malfunction or an adversarial attack. Fostering the development of AI in support of new abilities and skills, while anticipating and mitigating its impact on old ones will require both close study and potentially radical ideas, such as the proposal for some form of “universal basic income”, which is growing in popularity and experimental use. In the end, we need some intergenerational solidarity between those disadvantaged today and those advantaged tomorrow, to ensure that the disruptive transition between the present and the future will be as fair as possible, for everyone.

### What We Can Do: Enhancing Human Agency, Without Removing Human Responsibility

AI is providing a growing reservoir of “smart agency”. Put at the service of human intelligence, such a resource can hugely enhance human agency. We can do more, better, and faster, thanks to the support provided by AI. In this sense of “Augmented Intelligence”, AI could be compared to the impact that engines have had on our lives. The larger the number of people who will enjoy the opportunities and benefits of such a reservoir of smart agency “on tap”, the better our societies will be. Responsibility is therefore essential, in view of what sort of AI we develop, how we use it, and whether we share with everyone its advantages and benefits. Obviously, the corresponding risk is the absence of such responsibility. This may happen not just because we have the wrong socio-political framework, but also because of a “black box” mentality, according to which AI systems for decision-making are seen as being beyond human understanding, and hence control. These concerns apply not only to high-profile cases, such as deaths caused by autonomous vehicles, but also to more commonplace but still significant uses, such as in automated decisions about parole or creditworthiness.

Yet the relationship between the degree and quality of agency that people enjoy and how much agency we delegate to autonomous systems is not zero-sum, either pragmatically or ethically. In fact, if developed thoughtfully, AI offers the opportunity of *improving and multiplying* the possibilities for human agency. Consider examples of “distributed morality” in human-to-human systems such as peer-to-peer lending (Floridi 2013). Human agency may be ultimately supported, refined and expanded by the embedding of “facilitating frameworks”, designed to improve the likelihood of morally good outcomes, in the set of functions that we delegate to AI systems. AI systems could, if designed effectively, amplify and strengthen shared moral systems.

### What We Can Achieve: Increasing Societal Capabilities, Without Reducing Human Control

Artificial intelligence offers myriad opportunities for improving and augmenting the capabilities of individuals and society at large. Whether by preventing and curing diseases or optimising transportation and logistics, the use of AI technologies presents countless possibilities for reinventing society by radically enhancing what humans are collectively capable of. More AI may support better coordination, and hence more ambitious goals. Human intelligence augmented by AI could find new solutions to old and new problems, from a fairer or more efficient distribution of resources to a more sustainable approach to consumption. Precisely because such technologies have the potential to be so powerful and disruptive, they also introduce proportionate risks. Increasingly, we may not need to be either ‘in or on the loop’ (that is, as part of the process or at least in control of it), if we can delegate our tasks to AI. However, if we rely on the use of AI technologies to augment our own abilities in the wrong way, we may delegate important tasks and above all decisions to autonomous systems that should remain at least partly subject to human supervision and choice. This in turn may reduce our ability to monitor the performance of these systems (by no longer being ‘on the loop’ either) or preventing or redressing errors or harms that arise (‘post loop’). It is also possible that these potential harms may accumulate and become entrenched, as more and more functions are delegated to artificial systems. It is therefore imperative to strike a balance between pursuing the ambitious opportunities offered by AI to improve human life and what we can achieve, on the one hand, and, on the other hand, ensuring that we remain in control of these major developments and their effects.

### How We Can Interact: Cultivating Societal Cohesion, Without Eroding Human Self-Determination

From climate change and antimicrobial resistance to nuclear proliferation and fundamentalism, global problems increasingly have high degrees of coordination complexity, meaning that they can be tackled successfully only if all stakeholders co-design and co-own the solutions and cooperate to bring them about. AI, with its data-intensive, algorithmic-driven solutions, can hugely help to deal with such coordination complexity, supporting more societal cohesion and collaboration. For example, efforts to tackle climate change have exposed the challenge of creating a cohesive response, both within societies and between them. The scale of this challenge is such that we may soon need to decide between engineering the climate directly and designing societal frameworks to encourage a drastic cut in harmful emissions. This latter option might be undergirded by an algorithmic system to cultivate societal cohesion. Such a system would not be imposed from the outside; it would be the result of a self-imposed choice, not unlike our choice of not buying chocolate if we had earlier chosen to be on a diet, or setting up an alarm clock to wake up. “Self-nudging” to behave in socially preferable ways is the best form of nudging, and the only one that preserves autonomy. It is the outcome of human decisions and choices, but it can rely on AI solutions to be implemented and facilitated. Yet the risk is that AI systems may erode human self-determination, as they may lead to unplanned and unwelcome changes in human behaviours to accommodate the routines that make automation work and people’s lives easier. AI’s predictive power and relentless nudging, even if unintentional, should be at the service of human self-determination and foster societal cohesion, not undermining human dignity or human flourishing.

Taken together, these four opportunities, and their corresponding challenges, paint a mixed picture about the impact of AI on society and the people in it. Accepting the presence of trade-offs, seizing the opportunities while working to anticipate, avoid, or minimise the risks head-on will improve the prospect for AI technologies to promote human dignity and flourishing. Having outlined the potential benefits to individuals and society at large of an ethically engaged approach to AI, in the next section we highlight the “dual advantage” to organisations of taking such an approach.

## The Dual Advantage of an Ethical Approach to AI

Ensuring socially preferable outcomes of AI relies on resolving the tension between incorporating the benefits and mitigating the potential harms of AI, in short, simultaneously avoiding the misuse and underuse of these technologies. In this context, the value of an ethical approach to AI technologies comes into starker relief. Compliance with the law is merely necessary (it is the least that is required), but significantly insufficient (it is not the most than can and should be done) (Floridi 2018). With an analogy, it is the difference between playing according to the rules, and playing well, so that one may win the game. Adopting an ethical approach to AI confers what we define here as a “dual advantage”. On one side, ethics enables organisations to take advantage of the social value that AI enables. This is the advantage of being able to identify and leverage new opportunities that are socially acceptable or preferable. On the other side, ethics enables organisations to anticipate and avoid or at least minimise costly mistakes. This is the advantage of prevention and mitigation of courses of action that turn out to be socially unacceptable and hence rejected, even when legally unquestionable. This also lowers the opportunity costs of choices not made or options not grabbed for fear of mistakes.

Ethics’ dual advantage can only function in an environment of public trust and clear responsibilities more broadly. Public acceptance and adoption of AI technologies will occur only if the benefits are seen as meaningful and risks as potential, yet preventable, minimisable, or at least something against which one can be protected, through risk management (e.g. insurance) or redressing. These attitudes will depend in turn on public engagement with the development of AI technologies, openness about how they operate, and understandable, widely accessible mechanisms of regulation and redress. In this way, an ethical approach to AI can also be seen as an early warning system against risks that might endanger entire organisations. The clear value to any organisation of the dual advantage of an ethical approach to AI amply justifies the expense of engagement, openness, and contestability that such an approach requires.

## A Unified Framework of Principles for AI in Society

AI4People is not the first initiative to consider the ethical implications of AI. Many organisations have already produced statements of the values or principles that should guide the development and deployment of AI in society. Rather than conduct a similar, potentially redundant exercise here, we strive to move the dialogue forward, constructively, from principles to proposed policies, best practices, and concrete recommendations for new strategies. Such recommendations are not offered in a vacuum. But rather than generating yet another series of principles to serve as an ethical foundation for our recommendations, we offer a synthesis of existing sets of principles produced by various reputable, multi-stakeholder organisations and initiatives. A fuller explanation of the scope, selection and method of assessing these sets of principles is available in Cowls and Floridi (Forthcoming). Here, we focus on the commonalities and noteworthy differences observable across these sets of principles, in view of the 20 recommendations offered in the rest of the paper. The documents we assessed are:

1. The Asilomar AI Principles, developed under the auspices of the Future of Life Institute, in collaboration with attendees of the high-level Asilomar conference of January 2017 (hereafter “Asilomar”; Asilomar AI Principles 2017);
2. The Montreal Declaration for Responsible AI, developed under the auspices of the University of Montreal, following the Forum on the Socially Responsible Development of AI of November 2017 (hereafter “Montreal”; Montreal Declaration 2017)^3^;
3. The General Principles offered in the second version of *Ethically Aligned Design: A Vision for Prioritizing Human Well*-*being with Autonomous and Intelligent Systems*. This crowd-sourced global treatise received contributions from 250 global thought leaders to develop principles and recommendations for the ethical development and design of autonomous and intelligent systems, and was published in December 2017 (hereafter “IEEE”; IEEE 2017)^4^;
4. The Ethical Principles offered in the *Statement on Artificial Intelligence, Robotics and ‘Autonomous’ Systems,* published by the European Commission’s European Group on Ethics in Science and New Technologies, in March 2018 (hereafter “EGE”; EGE 2018);
5. The “five overarching principles for an AI code” offered in paragraph 417 of the UK House of Lords Artificial Intelligence Committee’s report, *AI in the UK: ready, willing and able?*, published in April 2018 (hereafter “AIUK”; House of Lords 2018); and
6. The Tenets of the Partnership on AI, a multistakeholder organisation consisting of academics, researchers, civil society organisations, companies building and utilising AI technology, and other groups (hereafter “the Partnership”; Partnership on AI 2018).

Taken together, they yield 47 principles.^5^ Overall, we find an impressive and reassuring degree of coherence and overlap between the six sets of principles. This can most clearly be shown by comparing the sets of principles with the set of four core principles commonly used in bioethics: beneficence, non-maleficence, autonomy, and justice. The comparison should not be surprising. Of all areas of applied ethics, bioethics is the one that most closely resembles digital ethics in dealing ecologically with new forms of agents, patients, and environments (Floridi 2013). The four bioethical principles adapt surprisingly well to the fresh ethical challenges posed by artificial intelligence. But they are not exhaustive. On the basis of the following comparative analysis, we argue that one more, new principle is needed in addition: *explicability*, understood as incorporating both intelligibility and accountability.

### Beneficence: Promoting Well-Being, Preserving Dignity, and Sustaining the Planet

Of the four core bioethics principles, beneficence is perhaps the easiest to observe across the six sets of principles we synthesise here. The principle of creating AI technology that is beneficial to humanity is expressed in different ways, but it typically features at the top of each list of principles. Montreal and IEEE principles both use the term “well-being”: for Montreal, “the development of AI should ultimately promote the well-being of all sentient creatures”; while IEEE states the need to “prioritize human well-being as an outcome in all system designs”. AIUK and Asilomar both characterise this principle as the “common good”: AI should “be developed for the common good and the benefit of humanity”, according to AIUK. The Partnership describes the intention to “ensure that AI technologies benefit and empower as many people as possible”; while the EGE emphasises the principle of both “human dignity” and “sustainability”. Its principle of “sustainability” represents perhaps the widest of all interpretations of beneficence, arguing that “AI technology must be in line with … ensur[ing] the basic preconditions for life on our planet, continued prospering for mankind and the preservation of a good environment for future generations”. Taken together, the prominence of these principles of beneficence firmly underlines the central importance of promoting the well-being of people and the planet.

### Non-maleficence: Privacy, Security and “Capability Caution”

Though “do only good” (beneficence) and “do no harm” (non-maleficence) seem logically equivalent, in both the context of bioethics and of the ethics of AI they represent distinct principles, each requiring explication. While they encourage well-being, the sharing of benefits and the advancement of the public good, each of the six sets of principles also cautions against the many potentially negative consequences of overusing or misusing AI technologies. Of particular concern is the prevention of infringements on personal privacy, which is listed as a principle in five of the six sets, and as part of the “human rights” principles in the IEEE document. In each case, privacy is characterised as being intimately linked to individuals’ access to, and control over, how personal data is used.

Yet the infringement of privacy is not the only danger to be avoided in the adoption of AI. Several of the documents also emphasise the importance of avoiding the misuse of AI technologies in other ways. The Asilomar Principles are quite specific on this point, citing the threats of an AI arms race and of the recursive self-improvement of AI, as well as the need for “caution” around “upper limits on future AI capabilities”. The Partnership similarly asserts the importance of AI operating “within secure constraints”. The IEEE document meanwhile cites the need to “avoid misuse”, while the Montreal Declaration argues that those developing AI “should assume their responsibility by working against the risks arising from their technological innovations”, echoed by the EGE’s similar need for responsibility.

From these various warnings, it is not entirely clear whether it is the people developing AI, or the technology itself, which should be encouraged not to do harm—in other words, whether it is Frankenstein or his monster against whose maleficence we should be guarding. Confused also is the question of intent: promoting non-maleficence can be seen to incorporate the prevention of both accidental (what we above call “overuse”) and deliberate (what we call “misuse”) harms arising. In terms of the principle of non-maleficence, this need not be an either/or question: the point is simply to prevent harms arising, whether from the intent of humans or the unpredicted behaviour of machines (including the unintentional nudging of human behaviour in undesirable ways). Yet these underlying questions of agency, intent and control become knottier when we consider the next principle.

### Autonomy: The Power to Decide (Whether to Decide)

Another classic tenet of bioethics is the principle of autonomy: the idea that individuals have a right to make decisions for themselves about the treatment they do or not receive. In a medical context, this principle of autonomy is most often impaired when patients lack the mental capacity to make decisions in their own best interests; autonomy is thus surrendered involuntarily. With AI, the situation becomes rather more complex: when we adopt AI and its smart agency, we *willingly* cede some of our decision-making power to machines. Thus, affirming the principle of autonomy in the context of AI means striking a balance between the decision-making power we retain for ourselves and that which we delegate to artificial agents.

The principle of autonomy is explicitly stated in four of the six documents. The Montreal Declaration articulates the need for a balance between human- and machine-led decision-making, stating that “the development of AI should *promote* the autonomy of all human beings *and control* … the autonomy of computer systems” (italics added). The EGE argues that autonomous systems “must not impair [the] freedom of human beings to set their own standards and norms and be able to live according to them”, while AIUK adopts the narrower stance that “the autonomous power to hurt, destroy or deceive human beings should never be vested in AI”. The Asilomar document similarly supports the principle of autonomy, insofar as “humans should choose how and whether to delegate decisions to AI systems, to accomplish human-chosen objectives”.

These documents express a similar sentiment in slightly different ways, echoing the distinction drawn above between beneficence and non-maleficence: not only should the autonomy of humans be promoted, but also the autonomy of machines should be restricted and made intrinsically reversible, should human autonomy need to be re-established (consider the case of a pilot able to turn off the automatic pilot and regain full control of the airplane). Taken together, the central point is to protect the intrinsic value of human choice—at least for significant decisions—and, as a corollary, to contain the risk of delegating too much to machines. Therefore, what seems most important here is what we might call “meta-autonomy”, or a “decide-to-delegate” model: humans should always retain the power to *decide which decisions to take*, exercising the freedom to choose where necessary, and ceding it in cases where overriding reasons, such as efficacy, may outweigh the loss of control over decision-making. As anticipated, any delegation should remain overridable in principle (deciding to decide again).

The decision to make or delegate decisions does not take place in a vacuum. Nor is this capacity to decide (to decide, and to decide again) distributed equally across society. The consequences of this potential disparity in autonomy are addressed in the final of the four principles inspired by bioethics.

### Justice: Promoting Prosperity and Preserving Solidarity

The last of the four classic bioethics principles is justice, which is typically invoked in relation to the distribution of resources, such as new and experimental treatment options or simply the general availability of conventional healthcare. Again, this bioethics principle finds clear echoes across the principles for AI that we analyse. The importance of “justice” is explicitly cited in the Montreal Declaration, which argues that “the development of AI should promote justice and seek to eliminate all types of discrimination”, while the Asilomar Principles include the need for both “shared benefit” and “shared prosperity” from AI. Under its principle named “Justice, equity and solidarity”, the EGE argues that AI should “contribute to global justice and equal access to the benefits” of AI technologies. It also warns against the risk of bias in datasets used to train AI systems, and—unique among the documents—argues for the need to defend against threats to “solidarity”, including “systems of mutual assistance such as in social insurance and healthcare”. The emphasis on the protection of social support systems may reflect geopolitics, insofar as the EGE is a European body. The AIUK report argues that citizens should be able to “flourish mentally, emotionally and economically alongside artificial intelligence”. The Partnership, meanwhile, adopts a more cautious framing, pledging to “respect the interests of all parties that may be impacted by AI advances”.

As with the other principles already discussed, these interpretations of what justice means as an ethical principle in the context of AI are broadly similar, yet contain subtle distinctions. Across the documents, justice variously relates to

- Using AI to correct past wrongs such as eliminating unfair discrimination;
- Ensuring that the use of AI creates benefits that are shared (or at least shareable); and
- Preventing the creation of *new* harms, such as the undermining of existing social structures.

Notable also are the different ways in which the position of AI, *vis*-*à*-*vis* people, is characterised in relation to justice. In Asilomar and EGE respectively, it is AI technologies themselves that “should benefit and empower as many people as possible” and “contribute to global justice”, whereas in Montreal, it is “the *development* of AI” that “should promote justice” (italics added). In AIUK, meanwhile, people should flourish merely “alongside” AI. Our purpose here is not to split semantic hairs. The diverse ways in which the relationship between people and AI is described in these documents hints at broader confusion over AI as a man-made reservoir of “smart agency”. Put simply, and to resume our bioethics analogy, are we (humans) the patient, receiving the “treatment” of AI, the doctor prescribing it? Or both? It seems that we must resolve this question before seeking to answer the next question of whether the treatment will even work. This is the core justification for our identification within these documents of a new principle, one that is not drawn from bioethics.

### Explicability: Enabling the Other Principles Through Intelligibility and Accountability

The short answer to the question of whether “we” are the patient or the doctor is that actually we could be either—depending on the circumstances and on who “we” are in our everyday life. The situation is inherently unequal: a small fraction of humanity is currently engaged in the design and development of a set of technologies that are already transforming the everyday lives of just about everyone else. This stark reality is not lost on the authors whose documents we analyse. In all, reference is made to the need to *understand* and *hold to account* the decision-making processes of AI. This principle is expressed using different terms: “transparency” in Asilomar; “accountability” in EGE; both “transparency” and “accountability” in IEEE; “intelligibility” in AIUK; and as “understandable and interpretable” for the Partnership. Though described in different ways, each of these principles captures something seemingly novel about AI: that its workings are often invisible or unintelligible to all but (at best) the most expert observers.

The addition of this principle, which we synthesise as “explicability” both in the epistemological sense of “intelligibility” (as an answer to the question “how does it work?”) and in the ethical sense of “accountability” (as an answer to the question: “who is responsible for the way it works?”), is therefore the crucial missing piece of the jigsaw when we seek to apply the framework of bioethics to the ethics of AI. It complements the other four principles: for AI to be beneficent and non-maleficent, we must be able to understand the good or harm it is actually doing to society, and in which ways; for AI to promote and not constrain human autonomy, our “decision about who should decide” must be informed by knowledge of how AI would act instead of us; and for AI to be just, we must ensure that the technology—or, more accurately, the people and organisations developing and deploying it—are held accountable in the event of a negative outcome, which would require in turn some understanding of why this outcome arose. More broadly, we must negotiate the terms of the relationship between ourselves and this transformative technology, on grounds that are readily understandable to the proverbial person “on the street”.

**Fig. 2:**
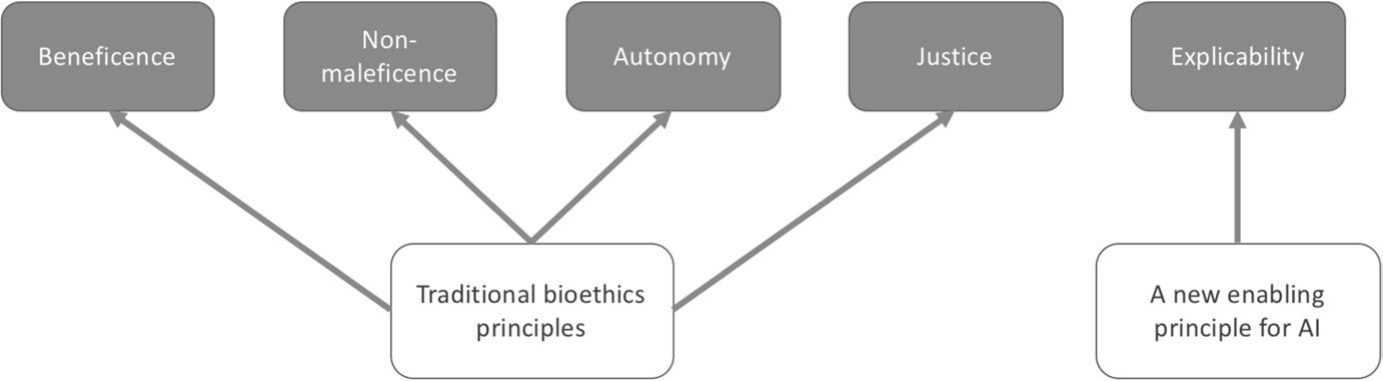
An ethical framework for AI, formed of four traditional principles and a new one

Taken together, we argue that these five principles capture the meaning of each of the 47 principles contained in the six high-profile, expert-driven documents, forming an ethical framework within which we offer our recommendations below. This framework of principles is shown in Fig. 2.

## Recommendations for a Good AI Society

This section introduces the Recommendations for a Good AI Society. It consists of two parts: a Preamble, and 20 Action Points.

There are four kinds of Action Points: to *assess,* to *develop,* to *incentivise* and to *support*. Some recommendations may be undertaken directly, by national or European policy makers, in collaboration with stakeholders where appropriate. For others, policy makers may play an enabling role for efforts undertaken or led by third parties.

### Preamble

We believe that, in order to create a Good AI Society, the ethical principles identified in the previous section should be embedded in the default practices of AI. In particular, AI should be designed and developed in ways that decrease inequality and further social empowerment, with respect for human autonomy, and increase benefits that are shared by all, equitably. It is especially important that AI be explicable, as explicability is a critical tool to build public trust in, and understanding of, the technology.

We also believe that creating a Good AI Society requires a multistakeholder approach, which is the most effective way to ensure that AI will serve the needs of society, by enabling developers, users and rule-makers to be on board and collaborating from the outset.

Different cultural frameworks inform attitudes to new technology. This document represents a European approach, which is meant to be complementary to other approaches. We are committed to the development of AI technology in a way that *secures people’s trust*, *serves the public interest,* and *strengthens shared social responsibility*.

Finally, this set of recommendations should be seen as a “living document”. The Action Points are designed to be dynamic, requiring not simply single policies or one-off investments, but rather, continuous, ongoing efforts for their effects to be sustained.

### Action Points

#### Assessment

1. *Assess* the capacity of existing institutions, such as national civil courts, to redress the mistakes made or harms inflicted by AI systems. This assessment should evaluate the presence of sustainable, majority-agreed foundations for liability from the design stage onwards, in order to reduce negligence and conflicts (see also Recommendation 5).^6^
2. *Assess* which tasks and decision-making functionalities should *not* be delegated to AI systems, through the use of participatory mechanisms to ensure alignment with societal values and understanding of public opinion. This assessment should take into account existing legislation and be supported by ongoing dialogue between all stakeholders (including government, industry, and civil society) to debate how AI will impact society opinion (in concert with Recommendation 17).
3. *Assess* whether current regulations are sufficiently grounded in ethics to provide a legislative framework that can keep pace with technological developments. This may include a framework of key principles that would be applicable to urgent and/or unanticipated problems.

#### Development

4. *Develop* a framework to enhance the explicability of AI systems that make socially significant decisions. Central to this framework is the ability for individuals to obtain a factual, direct, and clear explanation of the decision-making process, especially in the event of unwanted consequences. This is likely to require the development of frameworks specific to different industries, and professional associations should be involved in this process, alongside experts in science, business, law, and ethics.
5. *Develop* appropriate legal procedures and improve the IT infrastructure of the justice system to permit the scrutiny of algorithmic decisions in court. This is likely to include the creation of a framework for AI explainability as indicated in Recommendation 4, specific to the legal system. Examples of appropriate procedures may include the applicable disclosure of sensitive commercial information in IP litigation, and—where disclosure poses unacceptable risks, for instance to national security—the configuration of AI systems to adopt technical solutions by default, such as zero-knowledge proofs in order to evaluate their trustworthiness.
6. *Develop* auditing mechanisms for AI systems to identify unwanted consequences, such as unfair bias, and (for instance, in cooperation with the insurance sector) a solidarity mechanism to deal with severe risks in AI-intensive sectors. Those risks could be mitigated by multistakeholder mechanisms upstream. Pre-digital experience indicates that, in some cases, it may take a couple of decades before society catches up with technology by way of rebalancing rights and protection adequately to restore trust. The earlier that users and governments become involved—as made possible by ICT—the shorter this lag will be.
7. *Develop* a redress process or mechanism to remedy or compensate for a wrong or grievance caused by AI. To foster public trust in AI, society needs a widely accessible and reliable mechanism of redress for harms inflicted, costs incurred, or other grievances caused by the technology. Such a mechanism will necessarily involve a clear and comprehensive allocation of accountability to humans and/or organisations. Lessons could be learnt from the aerospace industry, for example, which has a proven system of handling unwanted consequences thoroughly and seriously. The development of this process must follow from the assessment of existing capacity outlined in Recommendation 1. If a lack of capacity is identified, additional institutional solutions should be developed at national and/or EU levels, to enable people to seek redress. Such solutions may include:
  An “AI ombudsperson” to ensure the auditing of allegedly unfair or inequitable uses of AI;
  A guided process for registering a complaint akin to making a Freedom of Information request; and
  The development of liability insurance mechanisms, which would be required as an obligatory accompaniment of specific classes of AI offerings in EU and other markets. This would ensure that the relative reliability of AI-powered artefacts, especially in robotics, is mirrored in insurance pricing and therefore in the market prices of competing products.^7^

Whichever solutions are developed, these are likely to rely on the framework for intelligibility proposed in Recommendation 4.

8. *Develop* agreed-upon metrics for the trustworthiness of AI products and services, to be undertaken either by a new organisation, or by a suitable existing organisation. These metrics would serve as the basis for a system that enables the user-driven benchmarking of all marketed AI offerings. In this way, an index for trustworthy AI can be developed and signalled, in addition to a product’s price. This “trust comparison index” for AI would improve public understanding and engender competitiveness around the development of safer, more socially beneficial AI (e.g., “IwantgreatAI.org”). In the longer term, such a system could form the basis for a broader system of certification for deserving products and services, administered by the organisation noted here, and/or by the oversight agency proposed in Recommendation 9. The organisation could also support the development of codes of conduct (see Recommendation 18). Furthermore, those who own or operate inputs to AI systems and profit from it could be tasked with funding and/or helping to develop AI literacy programs for consumers, in their own best interest.
9. *Develop* a new EU oversight agency responsible for the protection of public welfare through the scientific evaluation and supervision of AI products, software, systems, or services. This may be similar, for example, to the European Medicines Agency. Relatedly, a “post-release” monitoring system for AIs similar to, for example, the one available for drugs should be developed, with reporting duties for some stakeholders and easy reporting mechanisms for other users.
10. *Develop* a European observatory for AI. The mission of the observatory would be to watch developments, provide a forum to nurture debate and consensus, provide a repository for AI literature and software (including concepts and links to available literature), and issue step-by-step recommendation and guidelines for action.
11. *Develop* legal instruments and contractual templates to lay the foundation for a smooth and rewarding human–machine collaboration in the work environment. Shaping the narrative on the ‘Future of Work’ is instrumental to winning “hearts and minds”. In keeping with ‘A Europe that protects’, the idea of “inclusive innovation” and to smooth the transition to new kinds of jobs, a European AI Adjustment Fund could be set up along the lines of the European Globalisation Adjustment Fund.

#### Incentivisation

12. *Incentivise* financially, at the EU level, the development and use of AI technologies within the EU that are socially preferable (not merely acceptable) and environmentally friendly (not merely sustainable but favourable to the environment). This will include the elaboration of methodologies that can help assess whether AI projects are socially preferable and environmentally friendly. In this vein, adopting a ‘challenge approach’ (see DARPA challenges) may encourage creativity and promote competition in the development of specific AI solutions that are ethically sound and in the interest of the common good.
13. *Incentivise* financially a sustained, increased and coherent European research effort, tailored to the specific features of AI as a scientific field of investigation. This should involve a clear mission to advance AI for social good, to serve as a unique counterbalance to AI trends with less focus on social opportunities.
14. *Incentivise* financially cross-disciplinary and cross-sectoral cooperation and debate concerning the intersections between technology, social issues, legal studies, and ethics. Debates about technological challenges may lag behind the actual technical progress, but if they are strategically informed by a diverse, multistakeholder group, they may steer and support technological innovation in the right direction. Ethics should help seize opportunities and cope with challenges, not only describe them. It is essential in this respect that diversity infuses the design and development of AI, in terms of gender, class, ethnicity, discipline and other pertinent dimensions, in order to increase inclusivity, toleration, and the richness of ideas and perspectives.
15. *Incentivise* financially the inclusion of ethical, legal and social considerations in AI research projects. In parallel, incentivise regular reviews of legislation to test the extent to which it fosters socially positive innovation. Taken together, these two measures will help ensure that AI technology has ethics at its heart and that policy is oriented towards innovation.
16. *Incentivise* financially the development and use of lawfully de-regulated special zones within the EU for the empirical testing and development of AI systems. These zones may take the form of a “living lab” (or *Tokku*), building on the experience of existing “test highways” (or *Teststrecken*). In addition to aligning innovation more closely with society’s preferred level of risk, sandbox experiments such as these contribute to hands-on education and the promotion of accountability and acceptability at an early stage. “Protection by design” is intrinsic to this kind of framework.
17. *Incentivise* financially research about public perception and understanding of AI and its applications, and the implementation of structured public consultation mechanisms to design policies and rules related to AI. This may include the direct elicitation of public opinion via traditional research methods, such as opinion polls and focus groups, as well as more experimental approaches, such as providing simulated examples of the ethical dilemmas introduced by AI systems, or experiments in social science labs. This research agenda should not serve merely to measure public opinion, but should also lead to the co-creation of policies, standards, best practices, and rules as a result.

#### Support

18. *Support* the development of self-regulatory codes of conduct for data and AI related professions, with specific ethical duties. This would be along the lines of other socially sensitive professions, such as medical doctors or lawyers, i.e., with the attendant certification of ‘ethical AI’ through trust-labels to make sure that people understand the merits of ethical AI and will therefore demand it from providers. Current attention manipulation techniques may be constrained through these self-regulating instruments.
19. *Support* the capacity of corporate boards of directors to take responsibility for the ethical implications of companies’ AI technologies. For example, this may include improved training for existing boards and the potential development of an ethics committee with internal auditing powers. This could be developed within the existing structure of both one-tier and two-tier board systems, and/or in conjunction with the development of a mandatory form of “corporate ethical review board” to be adopted by organisations developing or using AI systems, to evaluate initial projects and their deployment with respect to fundamental principles.
20. *Support* the creation of educational curricula and public awareness activities around the societal, legal, and ethical impact of Artificial Intelligence. This may include:
  Curricula for schools, supporting the inclusion of computer science among the basic disciplines to be taught;
  Initiatives and qualification programmes in businesses dealing with AI technology, to educate employees on the societal, legal, and ethical impact of working alongside AI;
  A European-level recommendation to include ethics and human rights in the degrees of data and AI scientists and other scientific and engineering curricula dealing with computational and AI systems;
  The development of similar programmes for the public at large, with a special focus on those involved at each stage of management of the technology, including civil servants, politicians and journalists;
  Engagement with wider initiatives such as the ITU AI for Good events and NGOs working on the UN Sustainable Development Goals.

## Conclusion

Europe, and the world at large, face the emergence of a technology that holds much exciting promise for many aspects of human life, and yet seems to pose major threats as well. This article—and especially the Recommendations in the previous section—seek to nudge the tiller in the direction of ethically and socially preferable outcomes from the development, design and deployment of AI technologies. Building on our identification of both the core opportunities and the risks of AI for society as well as the set of five ethical principles we synthesised to guide its adoption, we formulated 20 Action Points in the spirit of collaboration and in the interest of creating *concrete* and *constructive* responses to the most pressing social challenges posed by AI.

With the rapid pace of technological change, it can be tempting to view the political process in the liberal democracies of today as old-fashioned, out-of-step, and no longer up to the task of preserving the values and promoting the interests of society and everyone in it. We disagree. With the Recommendations we offer here, including the creation of centres, agencies, curricula, and other infrastructure, we have made the case for an ambitious, inclusive, equitable programme of policy making and technological innovation, which we believe will contribute to securing the benefits and mitigating the risks of AI, for all people, and for the world we share.

## Footnotes

1 Besides Luciano Floridi, the members of the Scientific Committee are: Monica Beltrametti, Raja Chatila, Patrice Chazerand, Virginia Dignum, Christoph Luetge, Robert Madelin, Ugo Pagallo, Francesca Rossi, Burkhard Schafer, Peggy Valcke, and Effy Vayena. Josh Cowls is the rapporteur. Thomas Burri contributed to an earlier draft.

2 The analysis in this and the following two sections is also available in Cowls and Floridi (2018). Further analysis and more information on the methodology employed will be presented in Cowls and Floridi (Forthcoming).

3 The Montreal Declaration is currently open for comments as part of a redrafting exercise. The principles we refer to here are those which were publicly announced as of 1st May, 2018.

4 The third version of *Ethically Aligned Design* will be released in 2019 following wider public consultation.

5 Of the six documents, the Asilomar Principles offer the largest number of principles with arguably the broadest scope. The 23 principles are organised under three headings, “research issues”, “ethics and values”, and “longer-term issues”. We have omitted consideration of the five “research issues” here as they are related specifically to the practicalities of AI development, particularly in the narrower context of academia and industry. Similarly, the Partnership’s eight Tenets consist of both intra-organisational objectives and wider principles for the development and use of AI. We include only the wider principles (the first, sixth, and seventh tenets).

6 Determining accountability and responsibility may usefully borrow from lawyers in Ancient Rome who would go by this formula ‘cuius commoda eius et incommoda’ (‘the person who derives an advantage from a situation must also bear the inconvenience’). A good 2200 years old principle that has a well-established tradition and elaboration could properly set the starting level of abstraction in this field.

7 Of course, to the extent that AI systems are ‘products’, general tort law still applies in the same way to AI as it applies in any instance involving defective products or services that injure users or do not perform as claimed or expected.
